# Evolutionary Computation for Parameter Extraction of Organic Thin-Film Transistors Using Newly Synthesized Liquid Crystalline Nickel Phthalocyanine

**DOI:** 10.3390/mi10100683

**Published:** 2019-10-10

**Authors:** Juan A. Jiménez-Tejada, Adrián Romero, Jesús González, Nandu B. Chaure, Andrew N. Cammidge, Isabelle Chambrier, Asim K. Ray, M. Jamal Deen

**Affiliations:** 1 Departamento de Electrónica y Tecnología de los Computadores, Centro de Investigación en Tecnologías de la Información y de las Comunicaciones (CITIC), Universidad de Granada, 18071 Granada, Spain; tejada@ugr.es (J.A.J.-T.); ad90ry@correo.ugr.es (A.R.); 2 Departamento de Arquitectura y Tecnología de Computadores, Centro de Investigación en Tecnologías de la Información y de las Comunicaciones (CITIC), Universidad de Granada, 18071 Granada, Spain; jesusgonzalez@ugr.es; 3 Department of Physics, Savitribai Phule Pune University, Pune 411007, India; n.chaure@physics.unipune.ac.in; 4 School of Chemistry, University of East Anglia, Norwich Research Park, Norwich NR4 7TJ, UK; a.cammidge@uea.ac.uk (A.N.C.); i.fernandes@uea.ac.uk (I.C.); 5 Department of Electronic and Computer Engineering, Brunel University London, Uxbridge UB8 3PH, UK; Asim.Ray@brunel.ac.uk; 6 Department of Electrical and Computer Engineering, McMaster University, 1280 Main Street West, Hamilton, ON L8S 4K1, Canada; 7 Electronic Engineering, University of Electronic Science and Technology of China, Chengdu 611731, China

**Keywords:** contact effects, evolutionary multi-objective optimization, modeling, nickel phthalocyanine, thin-film transistor, device parameters

## Abstract

In this work, the topic of the detrimental contact effects in organic thin-film transistors (OTFTs) is revisited. In this case, contact effects are considered as a tool to enhance the characterization procedures of OTFTs, achieving more accurate values for the fundamental parameters of the transistor threshold voltage, carrier mobility and on-off current ratio. The contact region is also seen as a fundamental part of the device which is sensitive to physical, chemical and fabrication variables. A compact model for OTFTs, which includes the effects of the contacts, and a recent proposal of an associated evolutionary parameter extraction procedure are reviewed. Both the model and the procedure are used to assess the effect of the annealing temperature on a nickel-1,4,8,11,15,18,22,25-octakis(hexyl)phthalocyanine (NiPc_6_)-based OTFT. A review of the importance of phthalocyanines in organic electronics is also provided. The characterization of the contact region in NiPc_6_ OTFTs complements the results extracted from other physical–chemical techniques such as differential scanning calorimetry or atomic force microscopy, in which the transition from crystal to columnar mesophase imposes a limit for the optimum performance of the annealed OTFTs.

## 1. Introduction

Organic/polymeric electronic technologies have attracted considerable attention of both the academic and industrial research communities, due to their potential as a low cost, large area, solution-processable alternative to their conventional, inorganic counterparts [1,2,3,4]. In recent years, considerable interest has been shown in the development of active electronic devices such as photoconductors [5,6,7], chemiresistors [8,9] and the most popular organic thin-film field-effect transistors (OTFTs) [10,11].

OTFTs are not free of limitations, which makes this emerging technology advance at a slower pace than predicted. In this sense, recent advances were made to improve detrimental effects such as poor mobility [12,13,14,15], high process variability [16], non-flexible electrodes [17] or parasitic contact effects [18,19,20,21]. In parallel, many groups have been working in order to develop a universal compact model for OTFTs that incorporates the limitations and physical peculiarities of organic semiconductors [22,23,24,25,26,27,28,29,30,31,32,33,34]. Analyses of the features of different compact models were recently reviewed [35,36].

In this work, we stress on one of the above mentioned problems, the contact effects. We focus on two ways of taking advantage of the detrimental effect of the contact regions. The first of these is a way to improve the characterization of the device. Despite many research groups being aware that contact effects deteriorate the transistor behavior, on some occasions, these effects are not considered when extracting the values of the carrier mobility or threshold voltage from current-voltage characteristics. The complexity of the compact models or the parameter extraction procedures can lead researchers to use approximate models such as the crystalline field-effect-transistor (c-FET) model, obtaining important errors in the value of the device parameters. Here, we show an example of an easy-to-use and widely-employed [35,36,37,38,39,40,41,42,43] generic model for OTFTs [22,23], which allows a functional model of the carrier injection through the source contact.

Second, the contacts, like the intrinsic channel of the OTFT, are sensitive to changes in the environmental and fabrication conditions. It is well accepted that electronic characteristics such as charge carrier mobility, threshold voltage and on-off current ratio of OTFTs change upon the exposure of chemical or physical variables and are clearly dependent on the fabrication process. Thus, the determination of the values of such variables is a priority in order to improve a transistor design or to calibrate it to sense a particular physical or chemical variable [44,45,46]. Therefore, establishing an appropriate correlation between the contact region characteristics and the sensing or fabrication variables should also be a priority. It would add more valuable information on how the whole device reacts to changes in physical, chemical or fabrication variables. Thus, the knowledge and characterization of the contact region of an OTFT must run in parallel to the characterization of the typical parameters of the transistor such as the above mentioned carrier mobility, threshold voltage or on–off current ratio.

In Section 2, we describe the generic compact model for OTFTs we use throughout this work. In this section, we review how this model has evolved from its first proposal [22,23], towards its latest modifications [37,38,47]. We describe how it has been transformed, incorporating different characterization strategies or changing its functional appearance in order to gain versatility and to adapt it to different kinds of contacts.

In the last part of the paper, the model, in its latest form, is used in combination with an evolutionary parameter-extraction procedure to characterize and optimize -based OTFTs annealed at different temperatures. This particular case is used as a practical example to demonstrate how important the characterization of the contact region is. In this practical study, a fabrication variable, the annealing temperature, is assessed. The evolution of the current-voltage curves at the contact region as a function of the annealing temperature provides useful information that complements the results of other physical-chemical techniques such as atomic force micrographs or differential scanning calorimetry spectra. In Section 3, the state of the art of phthalocyanines, an important group of organic semiconductors used in the organic electronic devices, is reviewed. In Section 4, the experimental details are described, and in Section 5, the results are presented. Finally, the conclusions are provided in Section 6.

## 2. Compact Modeling and Evolutionary Parameter Extraction

### 2.1. Compact Model for the Characteristics of OTFTs

Compact models are analytical models that are able to reproduce the electrical behavior of a given device in all regions of operation. They must take into account any physical limitations and performance features of the device. This is the case of a widely-used generic model for OTFTs [22,23,35,36,37,38,39,40,41,42,43]:

(1) $$\begin{array}{ll} I_{D} & =k_{0}\frac{W}{L}\frac{[{\left(V_{G}-V_{T}-V_{S}\right)}^{\gamma +2}-\left(V_{G}-V_{T}-V_{D}{)}^{\gamma +2}\right]}{\gamma +2}\\ k_{0} & =\mu_{0}C_{ox} \end{array},$$

which includes the voltage drop at the source contact, $V_{S}\equiv V_{C}$, and electric field-dependent mobility [48],

(2) $$\mu =\mu_{0}{\left(V_{G}-V_{T}\right)}^{\gamma },$$

with $V_{T}$ being the threshold voltage, $C_{ox}$ the capacitance per unit area of the oxide, W and L the channel width and length, respectively, γ the mobility enhancement factor, and $\mu_{0}$ the mobility-related parameter, whose dimension is expressed as ${\mathrm{cm}}^{2}$/($V^{1+\gamma }$s). In order to provide a single value for the voltage dependent mobility, the mobility is evaluated at $V_{GT}=V_{G}-V_{T}=1$ V, thus $\mu (V_{GT}=1$ V) $=\mu_{0}$ in ${\mathrm{cm}}^{2}$/(Vs). The mobility enhancement factor γ is suggested to depend on the characteristic energy width $E_{0}=kT_{0}$ of an exponential tail distribution of the density of states (DOS):

(3) $$\gamma =2\frac{T_{0}}{T}-2=2\frac{E_{0}}{kT}-2,$$

where *k* is Boltzmann’s constant, T is the absolute temperature and $T_{0}$ is the specific equivalent absolute temperature that represents the steepness of the DOS exponential tail. Note that the model described with Equation (1) reduces to the simple c-FET model if contact effects are neglected ($V_{S}\equiv V_{C}=0$) and the carrier mobility is assumed as constant (γ=0, $\mu =\mu_{0}$):

(4) $$I_{D}=k_{0}\frac{W}{L}\left[\left(V_{G}-V_{T}\right)V_{D}-\frac{V_{D}^{2}}{2}\right],$$

The subthreshold regime was included in Equation (1) using an asymptotically interpolation function [22,23]:

(5) $$\begin{array}{ll} I_{D} & =k_{0}\frac{W}{L}\frac{V_{EODR}{\left(V_{S}\right)}^{\left(2+\gamma \right)}-V_{EODR}{\left(V_{D}\right)}^{\left(2+\gamma \right)}}{2+\gamma }\\ V_{EODR}\left(V\right) & =V_{SS}\ln\left[1+\exp\left(\frac{V_{G}-V_{T}-V}{V_{SS}}\right)\right] \end{array},$$

where $V_{SS}$ is a voltage parameter related to the steepness of the subthreshold characteristics of the Thin Film Transistor (TFT).

The analytical Equation (5) describes all operation modes of the transistor: triode, saturation, subthreshold and even reverse biasing. The model proposed in [22] also included other factors such as channel “length” modulation effects and threshold voltage sensitivity to bias voltages. In this work, they will not be considered as they cause tradeoffs during characterization [23]. Equation (5) also allows for a functional expression of the carrier current–voltage characteristics at the source contact region as $V_{S}\equiv V_{C}=V_{C}\left(I_{D};V_{G}\right)$. Note that Equation (1) considers that the voltage drop at the contacts of OTFTs is dominated by the source contact, as potentiometry measurements show [49].

In a recent review, different OTFT models were compared by using a particle swarm optimization (PSO) technique applied to experimental output characteristics [35]. The authors stated that the compact and comprehensive Marinov’s model [22,23] (5) was one of the best available models to predict the *I − V* characteristics of OFETs. In particular, they said, it was especially suited for characterizing short-channel devices. In this regard, past efforts were devoted to include in the model a proper analytical expression for the current–voltage curves at the contact region of the OTFT [40,50,51,52,53]. The objective of these studies was to reproduce different experimental behaviors observed at low drain voltages of the output characteristics of OTFTs. For contacts with low energy barriers, linear or quadratic behaviors can be observed [54,55,56]. For contacts with high energy barriers, a Schottky-like behavior is observed instead [51,53,57].

Independent researchers have developed different models for the contact region of OTFTs. If the carrier injection through the contact is space-charge limited, Equation (5) was redefined with the inclusion of a simple model for the contact region [50]:

(6) $$\begin{array}{l} I_{D}=M_{C}\times V_{C}{}^{m_{k}},\\ \forall m_{k}\in \mathbb{Z}:1\leq m_{k}\leq 2, \end{array}$$

where $m_{k}$ is a parameter that controls the shape, from linear to quadratic, of the $I_{D}-V_{C}$ contact curves. These different shapes can be observed in the triode region of the OTFTs, at low values of the drain voltage [54,55,56]. The parameter $M_{C}$ is related to the conductance of the contact region. For Ohmic contacts ($m_{k}=1$), $M_{C}$ coincides with the contact conductance, or the inverse of the contact resistance, $M_{C}=1/R_{C}$. Thus, $V_{S}=I_{D}R_{C}$. The Mott Gurney law is reproduced with $m_{k}=2$: $I_{D}=M_{C}V_{C}^{2}$.

The solution of the transport equations in a metal-organic structure lies underneath the analytical Equation (6). In a metal-organic structure, the current density j and the applied voltage $V_{C}$ are related as [58,59,60,61]:

(7) $$\begin{array}{l} V_{C}=\left(\frac{2}{3}\right){\left[\frac{2j}{\epsilon \mu \theta }\right]}^{1/2}\left[{\left(x_{c}+x_{p}\right)}^{3/2}-{\left(x_{p}\right)}^{3/2}\right]\\ x_{p}\equiv \frac{j\epsilon \theta }{2\mu {\left[\theta qp\left(0\right)\right]}^{2}};\;\;j=I_{D}/S, \end{array}$$

where qp(0) is the charge density at the metal–organic interface, θ is the ratio of free to total charge density, qθp is the free charge density, S is the cross section of the channel where the drain current $I_{D}$ flows, $x_{p}$ is a characteristic length defined as the point from the contact interface towards the organic film, at which the charge density $qp\left(x_{p}\right)$ decays to $qp\left(0\right)/\sqrt{2}$, ϵ is the organic dielectric constant and $x_{C}$ is the length of the contact region in the organic material. Depending on the relative values of $x_{p}$ and $x_{C}$, two well-known behaviors are inherent in Equation (7). If the characteristic length $x_{p}$ is a few times larger than the contact length $x_{C}$, Equation (7) shows an Ohmic behavior

(8) $$I_{D}\approx \frac{S\theta qp\left(0\right)\mu }{x_{C}}V_{C}\equiv \frac{1}{R_{C}}\times V_{C}^{1};$$

on the contrary, if $x_{p}$ is much smaller than $x_{C}$, Equation (7) shows a quadratic behavior [55,59]

(9) $$I_{D}\approx \frac{9\epsilon \theta \mu S}{8x_{C}^{3}}V_{C}^{2}\equiv M\times V_{C}^{2}.$$

Thus, the parameter $M_{C}$ can be defined for these two limit cases as $M_{C}=1/R_{C}$ for $m_{k}=1$ in Equation (8), and $M_{C}=M$ for $m_{k}=2$ in Equation (9). For $m_{k}=1$, the parameter $M_{C}$ coincides exactly with the conductance of the contact region, and thus, $M_{C}$ is proportional to the free charge density per unit area in the contact region $\sigma_{contact}$. The parameter $M_{C}$ was also demonstrated to be proportional to $\sigma_{contact}$ for $m_{k}>1$ [50]. Thus, whatever trend $\sigma_{contact}$ has with the gate voltage, the parameter $M_{C}$ will have the same trend, except for a multiplying factor. The value of $\sigma_{contact}$ is usually lower than the free charge density per unit area in the intrinsic channel $\sigma_{channel}$. Thus, we can write $\sigma_{contact}=\kappa \sigma_{channel}=\kappa \left(\frac{\varepsilon_{ox}}{t_{ox}}\right)\left(V_{G}-V_{T}\right)$ where κ is a proportionality constant *κ* ≤ 1, and *ε_ox_* and *t_ox_* are the dielectric constant and thickness of the gate insulator, respectively. The proportionality between the free charge densities at these two adjacent regions is assumed, since there is no physical reason to believe that the mobile charges in these two regions start appearing at very different gate voltages, or follow very different trends, unless local non-uniformities were present just at the contact region. Also, a dependence of $M_{C}$ with the mobility μ is expected, since Equation (7) explicitly depends on both the free charge density in the contact *qθp*(0) and the mobility *μ*. Considering the dependence of the mobility with ($V_{G}-V_{T}{)}^{\gamma }$ (Equation (2)) and the proportional relation between $\sigma_{contact}$ and $\left(V_{G}-V_{T}\right)$, then the parameter $M_{C}$ is expected to depend on the gate voltage as [50]:

(10) $$M_{C}=\alpha {\left(V_{G}-V_{T}\right)}^{1+\gamma },$$

where α is a proportionality constant. The subthreshold regime was also incorporated into Equation (10) by an asymptotic interpolation function [38]:

(11) $$M_{C}=\alpha V_{SS}\ln{\left[1+\exp\left(\frac{V_{G}-V_{T}}{V_{SS}}\right)\right]}^{1+\gamma }.$$

The Equation (10) in its whole range $\left(1\leq m_{k}\leq 2\right)$ has been checked experimentally in OTFTs that are free of hysteresis and free of local non-uniformities in the contact region [37,38,55,62,63,64]. Situations in which the parameter $M_{C}$ does not follow the Equation (10) indicate that a different behavior between the contact region and the active channel exists, such as a non-uniform distribution of traps or defects between both regions [63,64]. 

Similar functional dependences of the channel resistance and contact resistance with the mobility, and the mobility with the gate voltage, can be found in the literature [24,65]. Equation (10) is also valid for situations in which the value of the carrier mobility in the contact region $\mu_{0C}$ is different to that in the conducting channel $\mu_{0}$, although they can follow the same dependence with $V_{GT}$. Anisotropic conduction in the contact region can make the carrier mobility smaller in this region [66].

Thus, the variables $t_{\mathrm{ox}}$, $\varepsilon_{\mathrm{ox}}$ and $\mu_{0C}$ can appear in (10):

(12) $$M_{C}=\alpha^{\prime }\left(\varepsilon_{\mathrm{ox}}/t_{\mathrm{ox}}\right)\left(V_{G}-V_{T}\right)\mu_{0C}{\left(V_{G}-V_{T}\right)}^{\gamma },$$

where *α*′ is a proportionality constant. This model can explain the decrease of the value of the contact resistance observed in bottom-contact (BC) coplanar OTFTs when the gate-insulator thickness $t_{\mathrm{ox}}$ is reduced [67]. The parameter α’ carries information about the free to total charge carrier ratio *θ*, which is seen in Equations (7)–(9). This ratio can incorporate effects of traps or defects associated with the metal–semiconductor contact. Different kinds of traps or defects can be related to different fabrication factors that affect the electrode material, the semiconductor or the metal–organic interface [68], such as those produced by the annealing temperature. Another way of examining the effects of fabrication is by assessing the relation between $M_{C}$ and $\left(V_{G}-V_{T}\right)$. It must be noted that Equation (10) is deduced assuming $\sigma_{contact}=\kappa \sigma_{channel}$. Thus, if the values of the parameter $M_{C}$ extracted from experimental OTFT characteristics follow Equation (10), a proportional relation $\sigma_{contact}=\kappa \sigma_{channel}$ is guaranteed, meaning that traps or defects, if present, are uniformly distributed throughout the intrinsic channel and the contact region [50,62]. On the contrary, if the extracted values of $M_{C}$ do not follow Equation (10), this proportionality fails and the defects along the contact and intrinsic channel are expected to be non-uniformly distributed.

The value of the proportionality constant between the conductivity of these two regions κ, implicit in the value of α, was tested to be sensitive to the contact material [50], temperature [50] and illumination [38,69]. Recent studies on organic phototransistors (OPTs) have demonstrated that both photoconductive and photovoltaic effects impact not only the intrinsic region of the transistor but also the electrical behavior of the contact region [38]. On the one hand, the photovoltaic effect is present in Equation (10) through the variation of the threshold voltage $\Delta V_{T}$, which is usually modeled as [70,71]:

(13) $$V_{T}=V_{Td}-\Delta V_{T};\;\Delta V_{T}=\frac{AkT}{q}\ln\left(1+\frac{\eta qP}{I_{Dd}h\nu }\right),$$

where $V_{Td}$ and $I_{Dd}$ are the threshold voltage and the channel current at dark conditions, respectively, q is the magnitude of the electron charge, η is the quantum efficiency associated with the absorption process in the channel, hν is the photon energy and A is an empirical constant. On the other hand, the conductivity of the contact region was proposed to be affected by the photoconductive effect just like the conductivity of the bulk semiconductor. Experimentally, it was shown that the photocurrent in the off state $I_{B}$ is directly proportional to the incident light intensity P [72,73,74,75,76]:

(14) $$I_{B}=\beta_{I}P+I_{B_{0}},$$

where $I_{B_{0}}$ is the bulk current in dark and $\beta_{I}$ controls the linear increase of the bulk current with P. In this sense, the conductivity of the contact region should also be proportional to the incident light intensity P. Therefore, parameter α in Equation (10) was proposed and tested to be linearly dependent on P:

(15) $$\alpha =\beta_{\alpha }P+\alpha_{0},$$

where $\alpha_{0}$ is the value of α in dark and $\beta_{\alpha }$ controls the linear evolution of α with the light intensity.

In parallel to the studies of space-charge limited contacts, other researchers have worked on developing models for Schottky-limited contacts [47,51] and incorporating them into the generic compact model (5). In one of these models, the $I_{D}-V_{C}$ relation resembles a reverse-biased Schottky-diode [51]:

(16) $$I_{D}=-I_{0}\exp\left[{\left(\frac{V_{C}}{V_{0}}\right)}^{\sigma }\right]\left\{\exp\left[-\left(\frac{V_{C}}{V_{N}}\right)\right]-1\right\},$$

where $I_{0}$ is a gate-modulated diode-reverse-current, $V_{N}=\left(nkT\right)/q$ and n is the diode quality factor. The barrier lowering induced by the Schottky effect is assumed in the term ${\left(V_{C}/V_{0}\right)}^{\sigma }$. The Schottky effect, which depends on the electric field at the junction is assumed to be dependent on the contact voltage $V_{C}$, and $V_{0}$ and σ are fitting parameters [51]. Despite the resemblance of Equation (16) with the characteristic of a Schottky diode, the authors considered it just as a mathematical expression able to reproduce the $I_{D}-V_{C}$ curves and, consequently, the extracted diode parameters were considered as effective model parameters [52]. An equivalent empirical relation that reproduces Schottky-limited contacts with the same accuracy is [47]:

(17) $$\begin{array}{l} I_{D}=M_{C}\times V_{C}{}^{m_{k}}\\ \forall m_{k}\in \mathbb{Z}:0<m_{k}<1. \end{array}$$

Equations (6) and (17) have a similar appearance. However, Equation (6) is a concave function in the range $1\leq m_{k}\leq 2$, and Equation (17) is a convex function in the range $0<m_{k}<1$. This similarity made authors in [47] reformulate the current–voltage contact model (6) as:

(18) $$\begin{array}{l} I_{D}=M_{C}\times V_{C}{}^{m_{k}},\\ \forall m_{k}\in \mathbb{Z}:0\leq m_{k}\leq 2. \end{array}$$

Note that the use of model (18) in the range $1\leq m_{k}\leq 2$ has a physical background for space-charge limited contacts, as justified above. However, since there is no physical justification for Schottky-limited contacts, this results in a semi-empirical model. An interesting application of the reformulated model (18) was found in the characterization of an ammonia sensor. Modifications of the contact region were detected when the gas concentration varies, transforming the space-charge limited contact of a pristine OTFT into a Schottky barrier contact under exposure of the gas [47]. Under these circumstances, the use of a model that has a similar functional appearance in order to describe different kinds of contacts is a clear advantage in circuit simulation and device characterization. In the next section, the device characterization issue will be discussed. It begins with a brief description of different parameter extraction methods that have been used with model (5).

### 2.2. Parameter Extraction

The proposal of the generic model (5) [22] was accompanied with a parameter extraction method [23]. This method is based in the so-called $H_{VG}$ function [77], which extracts the values of γ and the threshold voltage $V_{T}$ from the linear regime of operation of the OTFT. The $H_{VG}$ function is the ratio of the integral of the drain current over the gate bias divided by the drain current. Subsequently, other alternatives were proposed to extract the parameters of single OTFTs operating in the linear regime, in which a proper sequence of mathematical functions derived from measured $I_{D}-V_{D}$ characteristics is necessary [78]. The clear advantage of these methods is the use of a single channel-length transistor to extract all the parameters of the transistor, including its contact region. Other techniques need the use of multiple transistors with different channel lengths in order to determine the contact resistance, such as the transmission line model (TLM) method [79,80]; or the voltage drop at the contact [40,51,81,82]. In some of these last cases, the current–voltage characteristics of the injecting contacts are extracted from the dependence of the conductance on channel length [81]. In other cases, the channel conductance of long-length channel devices is firstly determined from experimental transfer characteristics measured at low drain voltages. In long transistors, the gradual channel approximation is assumed to be valid. Then, the channel conductance is used to determine the voltage drop at the source contact in short channel devices [40,51].

The compact model (5) along with its associated parameter extraction method have been successfully used [35,50,55,62,64]. However, it was noticed that the $H_{VG}$ function does not always perform satisfactorily under the following conditions [83]:

- The extracted values of γ and $V_{T}$ are inaccurate if $V_{C}\neq 0$.
- As initially defined in [77], the integral in the function $H_{VG}$ must necessarily be evaluated from a gate voltage under the threshold voltage. Otherwise, the extracted values of γ and $V_{T}$ will differ from the actual ones.
- The integral in the function $H_{VG}$ performed with very few points (very few output characteristic curves) also leads to inaccurate values of γ and $V_{T}$. In these cases, the errors in the values of γ and $V_{T}$ can propagate through the next steps of the extraction procedure, providing a nonoptimized parameter set ($k_{0}$, γ, $V_{T},$ $m_{k}$, $M_{C}$).

In order to overcome these shortcomings, a multi-objective evolutionary parameter extraction procedure was proposed to simultaneously determine the parameters of both the compact model, defined with Equation (5), and the contact model, defined with Equation (6) [83], or its extension, defined with Equation (18) [47]. This evolutionary procedure accelerates the extraction process and provides higher precision values for these parameters. This procedure works in combination with an open source evolutionary tool called ECJ (A Java-based Evolutionary Computation Research System) [84].

The parameter extraction procedure is based on a search algorithm of the parameters of the combined Equations (5) and (18) that makes this model accurately fit experimental output characteristics of OTFTs ($I_{D_{i,j}}=I_{D}\left(V_{G_{i}},V_{D_{j}}\right)$, where i∈ℤ: 1≤i≤g and j∈ℤ: 1≤j≤d, and g and d are the total number of discrete values of $V_{G}$ and $V_{D}$, respectively). The evolutionary procedure uses only experimental output characteristics of OTFTs. Then, the results can be tested with experimental transfer characteristics. This evolutionary procedure ensures that the extracted parameters comply with the physical meaning on which they are based by adding rules in form of optimization objectives and constraints for the different parameters. The details of the evolutionary parameter extraction procedure were given in [37,83].

Briefly, we describe the four competing objectives of the many-objective optimization problem (MaOP) we formulate in this work:

- ($O_{1}$) to minimize the error between the experimental values of $I_{D_{i,j}}=I_{D}\left(V_{G_{i}},V_{D_{j}}\right)$ and their estimation $\hat{I_{D}}\left(V_{G_{i}},V_{D_{j}},x\right)$ from Equations (5),(18), where x refers to the set of parameters needed to compute Equations (5),(10) and (18) and x is defined as an individual of the population:
  (19) $$x=\left(k_{0},\gamma ,V_{T},V_{SS},m_{k},M_{C}\left(V_{G_{1}}\right),\ldots ,M_{C}\left(V_{G_{g}}\right)\right),$$
  where $x_{l}$ (l∈ℤ: 1≤l≤p) is a variable of an individual and p=g+5 is the length or number of variables of an individual (Equation (19));
- ($O_{2}$) to minimize the error between the voltage drops at the contact region $V_{S}\left(V_{G_{i}},V_{D_{j}},x\right)$ determined with Equation (18) and $\hat{V_{S}}\left(V_{G_{i}},V_{D_{j}},x\right)$, which is directly calculated from Equation (5) using the values of the experimental data ($I_{D_{i,j}}$, $V_{D_{j}}$, $V_{G_{i}}$) and the values of γ, $V_{T}$, $V_{SS}$ and $k_{0}$ determined in a previous iteration:
  (20) $$\begin{array}{ll} V_{S} & =V_{G}-V_{T}-V_{SS}\times \ln\left[\exp\left(\frac{{\left(\frac{I_{D}L\left(\gamma +2\right)}{Wk_{0}}+V_{EODR}{\left(V_{D}\right)}^{\left(\gamma +2\right)}\right)}^{\frac{1}{\gamma +2}}}{V_{SS}}\right)-1\right] \end{array};$$
  ($O_{3}$) to maximize the standard determination coefficient $R^{2}\left(V_{G_{i}},x\right)$ of the linear fit of $M_{C}{}^{1/\left(1+\gamma \right)}$ with $V_{G}$ extracted from Equation (12), or to minimize ($1-R^{2}\left(V_{G_{i}},x\right)$);
- and ($O_{4}$) to minimize the difference between $V_{T}$ [$x_{3}$ in (19)] and its estimation $\hat{V_{T}}\left(V_{G_{i}},x\right)$ extracted from the linear fit (12).

The normalized root-mean-square error (NRMSE) is used to quantify the objectives ($O_{1}$) and ($O_{2}$) [85]:

(21) $$\mathrm{NRMSE}\left(y,\hat{y}\right)=\sqrt{\frac{\sum_{z=1}^{w}{\left(y_{z}-{\hat{y}}_{z}\right)}^{2}}{\sum_{z=1}^{w}{\left(y_{z}-\bar{y}\right)}^{2}}},$$

where y represents the data set that we want to accurately approximate, $\hat{y}$ is the estimation of y, w is the number of data samples in y, and $\bar{y}$ is the mean value of the complete data set y.

Thus, the proposed MaOP, named O, consists of a set of four minimization objectives $O=\left(O_{1},O_{2},O_{3},O_{4}\right)$, where

(22) $$\begin{array}{ccc} O_{1}\left(x\right) & = & \mathrm{NRMSE}\left(I_{D}\left(V_{G_{i}},V_{D_{j}}\right),\hat{I_{D}}\left(V_{G_{i}},V_{D_{j}},x\right)\right),\\ O_{2}\left(x\right) & = & \mathrm{NRMSE}\left(V_{S}\left(V_{G_{i}},V_{D_{j}},x\right),\hat{V_{S}}\left(V_{G_{i}},V_{D_{j}},x\right)\right),\\ O_{3}\left(x\right) & = & 1-R^{2}\left(V_{G_{i}},x\right),\\ O_{4}\left(x\right) & = & \hat{V_{T}}\left(V_{G_{i}},x\right)-x_{3},\\ & & \forall i\in \mathbb{Z}:1\leq i\leq g,\forall j\in \mathbb{Z}:1\leq j\leq d. \end{array}$$

Objective ($O_{1}$) allows us to accurately reproduce the experimental output and transfer characteristics with the model defined with Equations (5) and (18), using the parameters coded in Equation (19). Objective ($O_{2}$) checks whether the trend of the $I_{D}-V_{C}$ curves extracted from Equation (20) along with the parameters coded in Equation (19) are physically valid. Objectives ($O_{3}$) and ($O_{4}$) are not mandatory [37] but serve as a guide in the search process to those solutions that approach to the trend given in Equation (10). However, in the case that the analyzed device is affected by a non-uniform distribution of traps or degradation-induced defects, the optimization of objectives ($O_{3}$) and ($O_{4}$) is not guaranteed [62]. In this case, objectives ($O_{3}$) and ($O_{4}$) should be removed, leaving the evolutionary procedure as it was originally defined in [37]. A more complete explanation of this evolutionary parameter extraction procedure can be found in [83]. In the rest of the work, we use the model defined with Equations (5) and (18) in combination with the just-described evolutionary parameter extraction procedure to assess the effect of the annealing temperature on phthalocyanine-based OTFTs. First, in the next section, we briefly describe the role phthalocyanines play in organic electronics.

## 3. Phthalocyanines

**Figure 1:**
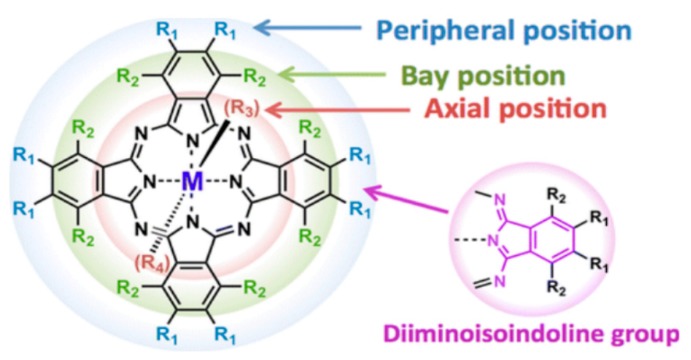
Phthalocyanine molecule.

Metal phthalocyanines (MPcs) are both thermally and chemically stable, non-toxic macrocyclic compounds containing conjugated cyclic π-systems of eighteen electrons in the ring compounds. As shown in Figure 1, the metal coordination is possible with any of up to 70 metal or metalloid elements at the isoindole nitrogen atoms with unique semiconducting properties offering tremendous scope of developing field-effect transistors with charge carrier mobility in the order of 1 ${\mathrm{cm}}^{2}V^{-1}s^{-1}$ [86,87]. The precursors for the synthesis of most MPcs are relatively inexpensive and can be obtained from major chemical manufacturers in large quantities. This consideration makes MPcs favorable for numerous commercial applications and is why numerous industrial laboratories are still exploring their use in a variety of applications. The majority of OTFTs have been p-type. However, an n-type MPc can be synthesized by attaching electron-withdrawing fluorines in all 16 peripheral and bay positions [88]. A p-type field-effect mobility of $5.4\times 10^{-4}$ ${\mathrm{cm}}^{2}V^{-1}s^{-1}$ was reported for thermally deposited a NiPc active layer at 300 °C in the bottom-gate bottom-gold-contact OTFT configuration on a $\mathrm{Si}/{\mathrm{SiO}}_{2}$ substrate [89]. Substitutions of aliphatic chains on sixteen sites available on the benzenoid rings confers solubility in conventional organic solvents including tetrahydrofuran, hydrocarbons such as toluene and chlorohydrocarbons such as dichloromethane and chloroform. The introduction of substituents onto the phthalocyanine nucleus can confer new and useful properties upon the ring system. These materials have proven to be ideal for deposition as well-ordered thin films by spin-coating as judged by low-angle X-ray reflectivity measurements.

For all these reasons, this class of solution processable, low-molecular-weight liquid crystalline phthalocyanine has become a candidate for the semiconducting layer in the design of OTFTs. In particular, phthalocyanine-based OTFTs can be found in a wide variety of sensing applications. NiPc-based organic photo field-effect transistors were demonstrated to be sensitive to humidity [90]. NiPc humidity sensors fabricated by drop-casting showed different sensitivity in room temperature capacitance and impedance variation when fabricated at different gravity conditions [91]. NiPc-based OTFTs are also sensitive to ${\mathrm{NO}}_{2}$. The current through a vacuum-sublimed NiPc film on silicon substrates was found to increase from $3\times 10^{-2}$ μA to 1.08 μA with increasing concentration of ${\mathrm{NO}}_{2}$ from 0 ppm to 160 ppm, while the sensitivity remained a constant of 0.94 within the concentration range between 10 ppm and 120 ppm [92]. Responses of a molecular material-based heterojunction consisting of hexadecafluorinated nickel phthalocyanine (Ni(F16Pc)) and nickel phthalocyanine (NiPc), (Au|Ni(F16Pc)|NiPc|Al) have been monitored by studying the current–voltage characteristics on exposure from an argon atmosphere to an ammonia-based one for three minutes. The value of the rectification ratio at ±10 V decreases from 520 for argon to 17 for ammonia [93].

In light-sensing applications, NiPc semiconductors are also present. A solution-processed red-sensitive organic photoconductive device was demonstrated by using soluble nickel phthalocyanine [Ni(t-Bu)4Pc]. The isomer ratio of Ni(t-Bu)4Pc with $C_{2\nu }$ was found to be an important factor to improve external quantum efficiency [94]. In addition, recent reports showed that the use of pristine vacuum thermally deposited NiPc as hole-transport-layer in perovskite-solar cells improves the performance of the cells when compared with other metal phthalocyanines such as CuPc: values of 14.3%, 19.45 mA ${\mathrm{cm}}^{-2}$, 1.0 V and 73.6% were obtained in the devices for maximum power conversion efficiency, short-circuit current density, open circuit voltage and fill-factor, respectively [95,96].

Other applications in which NiPc can be used include biomolecule sensors or water oxidation studies [97,98,99]. A simple chemically synthesized composite of 150-nm-wide nickel phthalocyanine (NiPc) nanofibers and reduced graphene oxide (rGO) exhibits a superior specific capacitance, 223.28 Fg^−1^ at 1 Ag^−1^, four-fold higher than the individual components, and also good stability. The interaction of NiPc nanofibres with the $H^{+}$ ions of the electrolyte on the rGO surface contribute to effective charge storage and delivery to the collecting electrode [97]. A nickel phthalocyanine (NiPc) nanofiber electrode was employed in ascorbic acid sensors in 0.2 M phosphate buffer over a wide concentration from 5.5 µM to 5.2 mM. The detection limit was 1.5 µM [98]. Thin films of novel noble-metal-free nickel phthalocyanine-based two-dimensional metal–organic frameworks deposited on fluorine-doped tin oxide glass substrates showed high catalytic oxygen evolution reaction activity with a very low onset potential (<1.48 V, overpotential < 0.25 V), high mass activity (883.3 Ag^−1^), and excellent catalytic durability. These observations appear to be very promising for water oxidation [99].

All these promising applications of NiPc semiconductors have caused researchers to thoroughly investigate the morphology of this material, since it determines the overall transistor performance in terms of the carrier mobility, threshold voltage and air stability. Ultrafast optical transient absorption and X-ray transient absorption spectroscopic studies of photoinduced axial ligation process in the excited state of nickel(II) octabutoxyphthalocyanine in pyridine solvent showed photoinduced axial ligation in its (d,d) excited state in the presence of ligating molecules [100]. The valence of the central metal atom, molecular weight, and the post-deposition conditions have influence on the morphology of the OTFT active layer [101].

Preliminary work on ${\mathrm{NiPc}}_{6}$ OTFTs showed that the grain boundaries in randomly oriented nano-rods in the ${\mathrm{NiPc}}_{6}$ films with a rough surface act as traps for charge carriers [102]. The existence of surface traps at grain boundaries can play a similar electrical role as interfaces in a transistor. The film of discotic tetra-substituted nickel phthalocyanine (${\mathrm{NiPcR}}_{4}$, $R=-\mathrm{SCH}{\left[{\mathrm{CH}}_{2}O{\left({\mathrm{CH}}_{2}{\mathrm{CH}}_{2}O\right)}_{2}C_{2}H_{5}\right]}_{2}$) deposited between two identical substrates is characterized by homeotropic alignment while no homeotropic alignments was obtained with an air interface. A similar behavior was observed for nickel phthalocyanine with a different substituent $R=-\mathrm{OCH}{\left({\mathrm{CH}}_{2}{\mathrm{OC}}_{12}H_{25}\right)}_{2}$ [103].

Another important aspect that can modify the morphology of the semiconducting material is the annealing temperature. Actually, the annealing temperature of the active layer of copper phthalocyanine [88] is found to have a significant effect on the performance of the OTFT. Molecules are well aligned in the spin-coated film with their columnar axis parallel to the substrate. The drain-source current is believed to be one-dimensional hole transport via the overlap of π−π molecular orbitals through the accumulation layer. The carrier mobility of the active layer of solution-processed ${\mathrm{SO}}_{3}$ Na-substituted NiPc annealed at 50 °C for 20 min is around 1 ${\mathrm{cm}}^{2}V^{-1}s^{-1}$ [104]. Room temperature conductivity of thin films of ${\mathrm{NiPcR}}_{4}$ between ITO and Al top contact is found to increase from 0.46 n$\Omega^{-1}$ $m^{-1}$ to 1.1 n$\Omega^{-1}$ $m^{-1}$ on annealing. The increase in conductivity is mainly due to the increased π−π interaction on changing from isotropic to columnar homeotropic alignment [105]. A reproducible shift of 4 nm in the surface plasmon resonance peak was observed when gold nanoparticles (AuNP) and discotic tetra-substituted nickel phthalocyanine (${\mathrm{NiPcR}}_{4}$, $R=-\mathrm{SCH}{\left[{\mathrm{CH}}_{2}O{\left({\mathrm{CH}}_{2}{\mathrm{CH}}_{2}O\right)}_{2}C_{2}H_{5}\right]}_{2}$) was transformed from hexagonal mesophase to isotropic liquid phase upon heating. Electrical conductivity of the composite is higher than that of the corresponding pure nickel phthalocyanine by two orders of magnitude [106].

In this context, it is important to link the electrical characteristics of the ${\mathrm{NiPc}}_{6}$ field-effect transistors and the morphological properties of the NiPc. In order to do this, in the next sections, we present a combined study of a thermal characterization of ${\mathrm{NiPc}}_{6}$ semiconductors with electrical characterization of OTFTs annealed at different temperatures. The thermal characterization is based on physical–chemical techniques such as differential scanning calorimetry spectra or atomic force micrographs. The electrical characterization is based on a compact model for OTFTs, which incorporates the effects of the contacts, combined with an evolutionary parameter extraction method.

## 4. Experimental Details

Bottom-gate, bottom-contact organic thin-film transistors (OTFTs) were fabricated using solvent soluble nickel-1,4,8,11,15,18,22,25-octakis(hexyl)phthalocyanine (${\mathrm{NiPc}}_{6}$) as the active semiconductor layer. These phthalocyanine compounds are known to exhibit thermotropic columnar liquid crystalline behavior. The purpose of the present work is, therefore, to explore the potential for optimization of the already promising properties of OTFTs using ${\mathrm{NiPc}}_{6}$ as the semiconducting layer via the use of thermal annealing [107].

The preparation of ${\mathrm{NiPc}}_{6}$ was straightforward, using the well-established sequence of macrocyclisation of phthalonitrile precursor (using lithium pentoxide in pentanol) followed by insertion of nickel. We have used a highly doped n-type <001> crystalline silicon (resistivity 0.1 to 0.5 Ω/cm) as a substrate, which acts as the gate electrode. A 200-nm-thick ${\mathrm{SiO}}_{2}$ layer, having capacitance per unit area $C_{i}=9$ nF/${\mathrm{cm}}^{2}$, was grown thermally onto the Si substrate, which prevents the leakage between gate and source/drain electrodes. Prior to growing the ${\mathrm{SiO}}_{2}$ layer the substrates were cleaned ultrasonically with acetone, iso-propanol and double distilled water for 10–15 min each. The multistage spin coater, Model, KW-4A (Chemat Technology Inc.) was employed for the spin coating of ${\mathrm{NiPc}}_{6}$. A uniformly covered 70 nm ${\mathrm{NiPc}}_{6}$ layer was spun coated initially at spin speed 1000 rpm for 15 s and subsequently 3000 rpm for 60 s onto the ${\mathrm{SiO}}_{2}$ gate dielectric. A ${\mathrm{NiPc}}_{6}$ solution was prepared in toluene with the concentration of 10 mg in 1 mL. The photolithographic and sputtering techniques were used to prepare the finger-shaped gold electrodes consisting of 10 nm Ti and 50 nm Au, which serve as source and drain electrodes. The back side of the Si wafer was removed and a layer of Ga–In eutectic was applied in order to form the Ohmic contact and was used as a gate terminal. The resulting inverted structure of the OTFTs had a channel length L=20 μm and a channel width W=10 mm. A DekTak profilometer was employed to measure the film thickness. The active layer of the transistors was ex-situ vacuum annealed at temperatures between 50 °C and 150 °C. The electrical measurements on the as-prepared and annealed ${\mathrm{NiPc}}_{6}$ OFETs were performed at room temperature under ambient conditions, using a Keithley 4200 semiconductor parameter analyzer. Further details can be obtained from an earlier publication [108].

Heat treatments of the ${\mathrm{NiPc}}_{6}$ semiconductor were conducted. The transitions induced due to heating and the subsequent cooling re-crystallization were recorded using differential scanning calorimetry (DSC), model Perkin-Elmer Pyris DSC-7. A specially designed aluminum (Al) pan was used for the DSC measurements. A powder of ${\mathrm{NiPc}}_{6}$ (10 mg) was sealed in the Al pan at a desired pressure. The multiple measurements (both exothermic and endothermic) were performed for 10 °C/min. DSC results show that only a single crystal morphology is formed in all cases. The observed effects are therefore interpreted as macroscopic and relate to crystal size and ordering. The surface morphology of the spin-coated ${\mathrm{NiPc}}_{6}$ thin film was investigated using the Digital Nanoscope III, atomic force microscope (AFM). A cantilever tip (Silicon, NSC15/AIBS, Mikromasch, force constant k = 46 N/m) with cantilever resonance frequency at 390 kHz was used as the measuring probe. High resolution images of 512 × 512 pixels with a scan size of 5 × 5 $\mu m^{2}$ were obtained. All measurements were repeated on similarly prepared samples with a view to examine the reproducibility of the results.

## 5. Results and Discussion

Experimental results are presented along with their interpretation in both ${\mathrm{NiPc}}_{6}$ semiconductors and ${\mathrm{NiPc}}_{6}$ OTFTs. The objective is to identify the mechanisms responsible for the morphological changes produced in the semiconductor during heating treatments, and in what way they affect the electrical performance of OTFTs annealed at different temperatures. The results of DSC, AFM and electrical characterization are presented separately. Finally, a discussion of the combined results is provided.

### 5.1. Differential Scanning Calorimetry (DSC)

**Figure 2:**
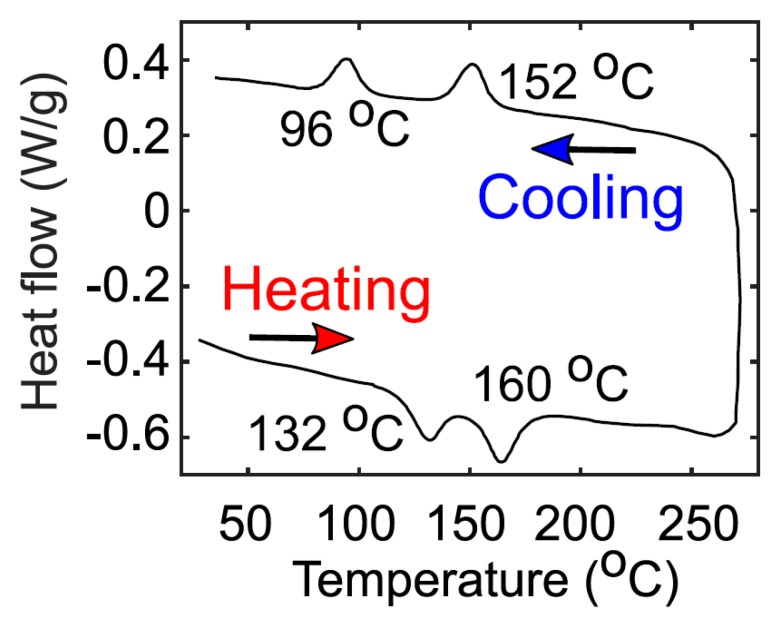
Differential scanning calorimetry of pristine ${\mathrm{NiPc}}_{6}$ powder for both heating (endothermic) and cooling (exothermic). The powder is collected from a spin-coated sample.

The first heating (endothermic) and cooling (exothermic) DSC spectra of pristine ${\mathrm{NiPc}}_{6}$ powder is depicted in Figure 2. The endothermic curve exhibits two thermally induced melts at about 132 and 160 °C, which are associated to the presence of several polymorphs. The initial peak is attributed to the transition of crystal to columnar mesophase, whereas the second is associated with mesophase to isotropic liquid transition. The two transitions were again detected in the exothermic measurements at about 96 and 152 °C, which are related to the re-crystallization of ${\mathrm{NiPc}}_{6}$ [109].

### 5.2. Atomic Force Microscope (AFM)

**Figure 3:**
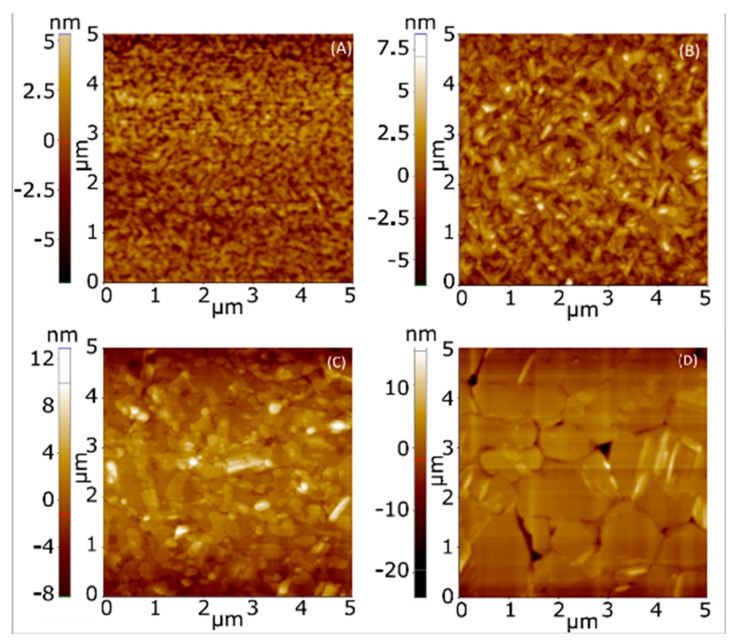
Two-dimensional AFM micrographs of the surfaces of the thin films of ${\mathrm{NiPc}}_{6}$: (**A**) as-prepared, prior to heat treatment; heat treatment during two hours at (**B**) 50 °C, (**C**) 100 °C and (**D**) 150 °C.

The surface topographical images of as-prepared and heat-treated ${\mathrm{NiPc}}_{6}$ thin layers spin-coated over ${\mathrm{SiO}}_{2}$ layer were taken with an atomic force microscope. The two-dimensional AFM images of as-prepared and heat-treated layers at 50, 100 and 150 °C are shown in Figure 3a–d, respectively. It is noteworthy that the growth of all samples was compact, well adherent, void free, and grains are well connected to each other. A mixed, granular and long grain-like morphology with random orientation of size over 200 nm is observed for the as-prepared sample. Upon the heat treatment the particle size is found to be increased (Table 1). The sample treated at 100 °C shows a clear enhancement in the particle size. The length of the rods (≈500 nm), along with small arbitrary shaped particles, can be seen in Figure 3c. It can be further seen that the orientation of the rods is random and the full surface of substrate is covered without voids or pinholes. Large disc-shaped sheets of size ranging from 1 to 2 μm are obtained upon annealing the samples at 150 °C (Figure 3d). We noticed that the layer was very compact, well adherent to the substrate. Upon increasing the heating temperature beyond 150 °C, we observed large chunks of materials with huge voids, may be due to the sublimation of ${\mathrm{NiPc}}_{6}$. For this reason, measurements above 150 °C were not performed.

### 5.3. Electrical Behavior of $NiPc_{6}$-Based OTFTs

#### 5.3.1. Current-Voltage Curves

**Figure 4:**
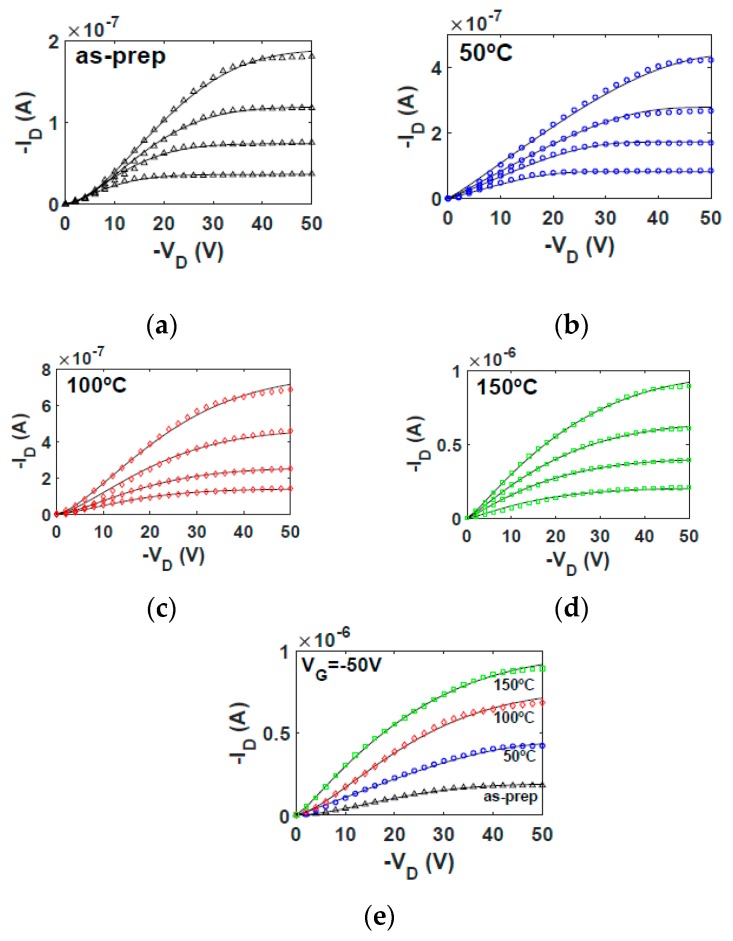
Output characteristics of ${\mathrm{NiPc}}_{6}$ organic thin-film transistors (OTFTs) at different annealing temperatures. (**a**–**d**) Comparison of experimental $I_{D}-V_{D}$ curves (symbols) and our calculations using Equations (5) and (18) (solid lines) at different annealing temperatures: (**a**) as-prepared; (**b**) 50 °C; (**c**) 100 °C; and (**d**) 150 °C. $V_{G}$ is swept from −20 (bottom) to −50 V (top) with a −10 V step. (**e**) Output characteristics at $V_{G}$ = −50 V of transistors annealed at different temperatures.

**Figure 5:**
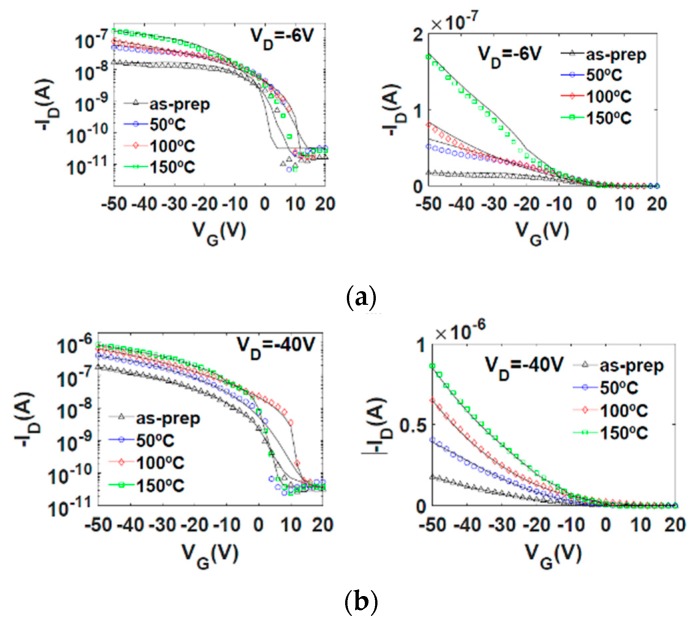
Comparison of experimental (symbols) and calculated (solid lines) transfer characteristics at different drain voltages $V_{D}$ in ${\mathrm{NiPc}}_{6}$ OTFTs annealed at different temperatures: (**a**) $V_{D}$ = −6 V; (**b**) $V_{D}$ = −40 V. Note that the left side figures contain the same $I_{D}$ values as the right side figures, but with different scale (logarithmic and linear).

The electrical current–voltage characteristics of the as-prepared and annealed OTFTs are shown in Figure 4 and Figure 5. Figure 4 shows, with symbols, the output characteristics measured at different values of the gate voltage $V_{G}$. Figure 5 shows, with symbols, the transfer characteristics measured in the linear ($V_{D}$= −6 V) and saturation ($V_{D}$= −40 V) regions. Figure 4e shows a comparison of experimental output characteristics (symbols) measured in the four transistors at the same gate voltage ($V_{G}$ = −50 V). At first glance, the higher the annealing temperature, the more the drain current is enhanced. A similar trend is observed in the on–off current ratio, which improves with the thermal annealing.

The objective of this study is to determine the origin of such increments of the drain current and on–off current ratio with the annealing temperature and to establish a connection between the electrical changes observed in Figure 4 and Figure 5 and the morphological and structural changes of the (${\mathrm{Ni}}_{6}\mathrm{Pc}$) semiconductor observed in Figure 2 and Figure 3. The current flowing through the active layer of an OTFT can be controlled by different factors. Some of them appear explicitly in the analytical expressions that reproduce the current–voltage curves of the OTFTs, such as the threshold voltage, which gives information about the availability of free charge carriers in the channel at a certain gate voltage; or the mobility of the charge carriers, which gives information about how the charge carriers respond to an applied electric field. There are other implicit factors that can modify the drain current through the OTFT, such as the contact effects, which can reduce the value of the drain current, and if they are not taken into account in the models, errors in the intrinsic parameters could be produced. For this reason, a compact model for the current–voltage characteristics of OTFTs that includes the modeling of the contact effects is necessary. The model must reproduce the electrical characteristics of the OTFTs, and the parameters of the model for this reproduction to occur must be extracted. Subsequently, the evolution of the different parameters of the model with the annealing temperature must be assessed.

#### 5.3.2. Evolution of OTFT Parameters with Annealing Temperature

**Figure 6:**
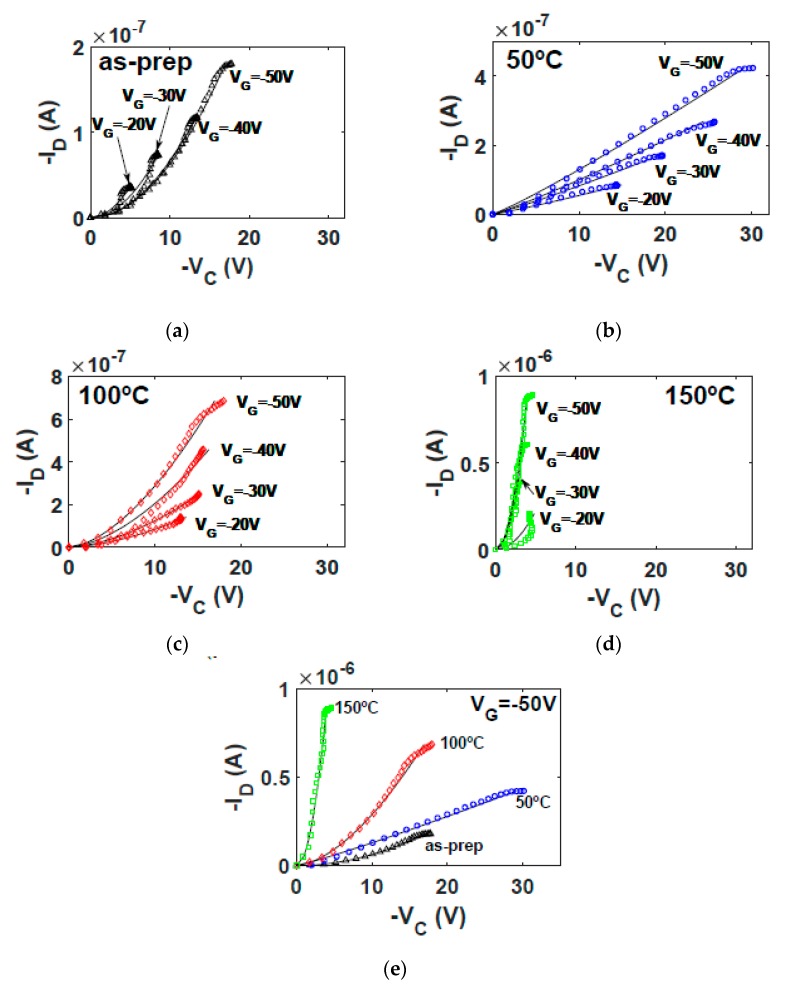
Contact $I_{D}-V_{C}$ curves in ${\mathrm{NiPc}}_{6}$ OTFTs at different annealing temperatures. (**a**–**d**) Comparison of $I_{D}-V_{C}$ curves calculated with Equation (20) (symbols) and Equation (18) (solid lines) at different annealing temperatures: (**a**) as-prepared; (**b**) 50 °C; (**c**) 100 °C; and (**d**) 150 °C. (**e**) Comparison of $I_{D}-V_{C}$ curves calculated with Equation (20) (symbols) and Equation (18) (solid lines) at $V_{G}$ = −50 V and different annealing temperatures.

In order to determine how the values of the model parameters evolve with temperature, the evolutionary procedure is independently applied to each experimental $I_{D}-V_{D}$ curve set of Figure 4a–d (symbols). The values of the parameters that make Equations (5) and (18) accurately fit experimental output characteristics are in Table 1. The $I_{D}-V_{D}$ curves calculated with Equations (5) and (18) are represented with solid lines in Figure 4. The good agreement between experimental and calculated data indicate that the first objective of the evolutionary algorithm is successfully achieved. The second objective is also achieved, as can be seen in Figure 6, in which a good agreement between the $I_{D}-V_{C}$ curves calculated independently with Equations (18) and (20) is observed (solid lines and symbols, respectively). In addition, a very good agreement is also observed in Figure 5 between experimental transfer characteristics (symbols) and our calculations (solid lines). Next, we analyze the evolution with the annealing temperature of the parameters of the Equations (5) and (18).

The evolution with the annealing temperature of the $I_{D}-V_{C}$ curves calculated at $V_{G}=-50$ V is seen in Figure 6e. This figure shows how the voltage drop at the contact is reduced when the annealing temperature is increased. This increase is not gradual since the largest increase is observed at 100 °C. The curvature of the $I_{D}-V_{C}$ curves also evolves with the annealing, changing from quadratic (as-prepared) to linear (50 °C), and again recovering the quadratic shape from 100 °C to 150 °C. The values of the parameter $m_{k}$ are 1.98, 1.10, 1.64 and 2.00 for the as-prepared, 50 °C, 100 °C and 150 °C annealing temperatures, respectively. Note that the exponent $m_{k}$ in Equation (6) controls the shape of the $I_{D}-V_{C}$ curves. This means that the way the free charges are transported in the contact region also changes, alternating from I−V characteristics that follows the Mott Gurney law to an almost Ohmic behavior as seen at 50 °C. These changes in the slope of the $I_{D}-V_{C}$ curves at the contact are related to the relative values of the contact length $x_{C}$ and the characteristic length $x_{p}$, both defined in Equation (7). In the as-prepared and 150 °C cases, $m_{k}≃2$ means that $x_{p}\ll x_{C}$; thus, the charge is concentrated close to the interface between electrode and semiconductor. At 50 °C, corresponding to the Ohmic case ($x_{p}\gg x_{C}$), the charge penetrates further inside the organic material. The 100 °C case corresponds to the transition between these two cases. These changes in the distribution of charge along the contact region might be originated by structural changes produced in the semiconductor when annealed at these temperatures and detected in the DSC spectra (Figure 2).

**Figure 7:**
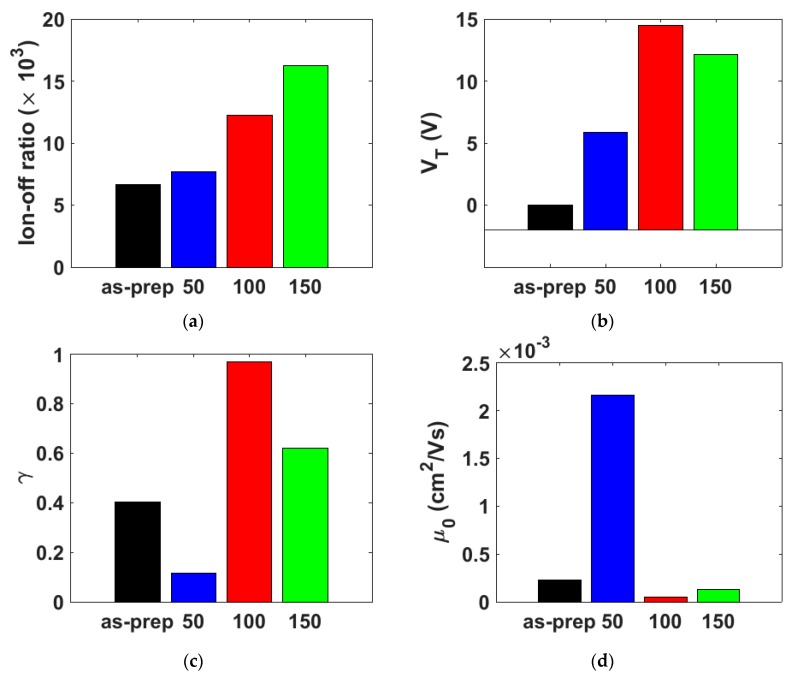
Comparative study of the annealed devices. (**a**) On-off current ratio; (**b**) threshold voltage $V_{T}$; (**c**) γ values; (**d**) mobility evaluated at $V_{GT}=V_{G}-V_{T}=1\;V$ [$\mu (V_{GT}=1\;V){=}_{0}$ in ${\mathrm{cm}}^{2}V^{-1}s^{-1}$].

The evolution of the threshold voltage $V_{T}$ with the annealing temperature (Figure 7b) also shows a non-monotonic trend. It increases toward more positive values, from around 0 V in the as-prepared transistor to 5.9 V at 50 °C, and to 14.5 V at 100 °C. However, the threshold voltage weakly decreases its value to 12.1 V at 150 °C.

**Figure 8:**
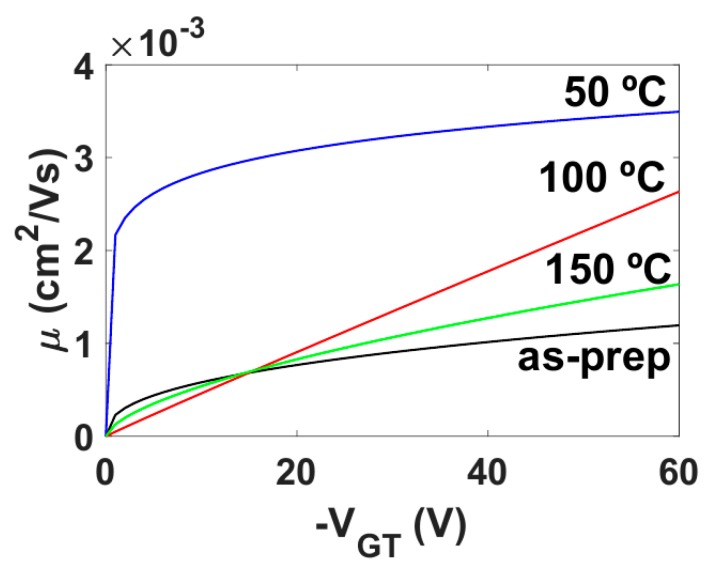
Mobility calculated using Equation (2) for different $V_{GT}$ values at different annealing temperatures.

The evolution with the annealing temperature of the parameters γ and $\mu_{o}$ are represented in Figure 7c,d, respectively. The values of the carrier mobility at different gate voltages are determined by including the values of $\mu_{o}$, $V_{T}$ and γ in Equation (2). The evolution with the annealing temperature of the carrier mobility at different values of $V_{GT}=V_{G}-V_{T}$ is represented in Figure 8. Overall, the mobility is highly influenced by the annealing temperature. Initially, there is a one-order of magnitude increase at 50 °C, in the whole range of $V_{GT}$. The greatest value of $\mu_{o}\;(V_{GT}$ = 1 V) is obtained at 50 °C as seen in Figure 7d. At 100 °C, and then at 150 °C, the values of the mobility diminish and converge to the values of the as-prepared samples. However, this convergence depends on the value of $V_{GT}$. At low values of $V_{GT}$, the convergence is faster than at high values of $V_{GT}$. The mobility enhancement factor γ, which is described by Equation (3), controls the dependence of the mobility with the electric field, and it is associated to the width of a band tail distribution of the density of states (DOS). Note that a low value of γ (γ→0) may be an indicator of crystalline-like behavior. The lowest value is γ=0.116 for 50 °C. This agrees with the 132 °C and 96 °C peaks in the DSC spectrum during heating (transition from crystal to columnar mesophase) and cooling (re-crystallization), respectively. The 50 °C case would lie in the crystal phase range. The evolution of γ with the annealing temperature is also consistent with the evolution of $m_{k}$. The Ohmic conduction detected at 50 °C coincides with the increase of the mobility and the decrease of γ. Thus, variations of this factor γ with the annealing temperature can be an indicator of variations in the electronic potential throughout the organic semiconductor produced by structural changes in the semiconductor. 

**Figure 9:**
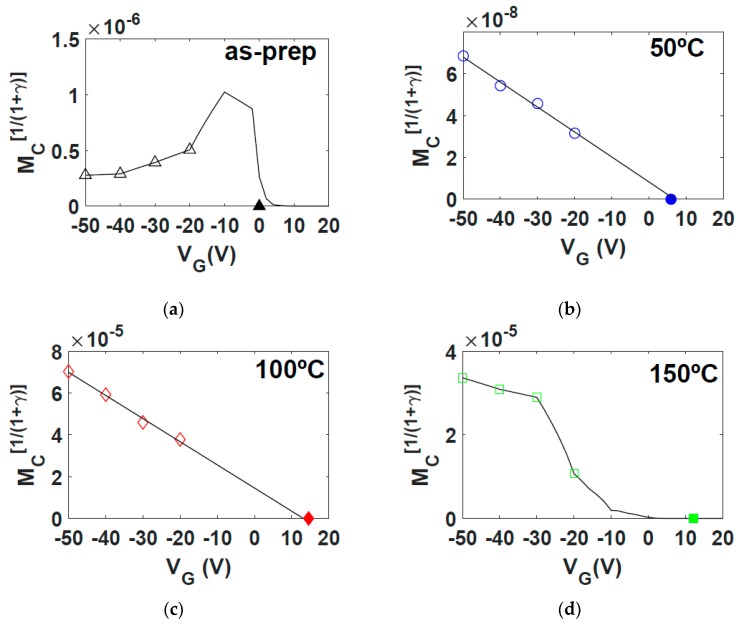
(**a**–**d**) Extracted values of $M_{C}{\left(V_{Gi}\right)}^{1/\left(1+\gamma \right)}$ (empty symbols) at different annealing temperatures. The solid lines show the trend that $M_{C}$ must follow for the Equations (5) and (18) to fit the experimental $I_{D}-V_{G}$ curves of Figure 5. In (**b**,**c**), the lines also follow the trend given in Equation (10). The black symbol drawn on the $V_{G}$ -axis of each figure represents the value of $V_{T}$ extracted with the evolutionary procedure.

Finally, the values of $M_{C}$, which depend on the gate voltage, are represented in Figure 9. The empty symbols are the values extracted with the evolutionary procedure from the experimental output characteristics (Figure 4). The solid lines represent the values of $M_{C}$ that must be introduced in the Equations (5) and (18) in order to fit the experimental $I_{D}-V_{G}$ curves of Figure 5. Clearly, only at 50 and 100 °C, the relation between $M_{C}$ and $V_{G}$ follows the trend given in Equation (12). Note for these two cases that the intercept values between the two lines and the $V_{G}$-axis (${\hat{V}}_{T}$) are close to the values of the extracted $V_{T}$ (drawn with black symbols on the $V_{G}$-axis of the respective figures; also written in Table 1). On the contrary, in the as-prepared and 150 °C cases, the extracted values of $M_{C}{\left(V_{G_{i}}\right)}^{1/\left(1+\gamma \right)}$ cannot be fitted with the Equation (10). However, the solid lines shown in Figure 7a and Figure 9d, which correspond to the values of $M_{C}$ used in the fitting of the experimental transfer characteristics (Figure 5), also intercept the $V_{G}$-axis close to the values of the extracted $V_{T}$. The deviation from this linear trend can be explained by an irregular distribution of traps or structural defects along the contact region and the adjacent intrinsic channel (Section 2.1). From this analysis, we can deduce that the as-prepared ${\mathrm{NiPc}}_{6}$ transistor is affected by these irregularities. After the annealing between 50 and 100 °C, the degrading effects are significantly reduced or uniformly redistributed throughout contact region and intrinsic channel. The non-uniformity between contact region and intrinsic channel appear again when the transistor is annealed at 150 °C. In any case, the intercept of all the four solid lines close to the values of the extracted $V_{T}$ means that the free charges along the active channel and along the contact region, although different in concentration, start appearing at the same gate voltage. Note that the detection of these irregularities in the contact region in the as-prepared and 150 °C annealed samples is extracted only from the analysis of the I−V characteristics, which clearly complements the DSC and AFM studies.

#### 5.3.3. Discussions

The clearest conclusion after analyzing the current–voltage curves of the ${\mathrm{NiPc}}_{6}$ OTFTs is that the drain current (Figure 4e) and the on–off current ratio (Figure 7a) increase monotonically with the annealing temperature, up to the maximum value used in the experiments (150 °C). However, this fact does not mean that the best performance is achieved at the highest annealing temperature, since the physical mechanisms that take place inside the transistor do not follow the same trend. This can be seen if the evolution with the annealing temperature of the different parameters that appear in the transistor model is analyzed.

The relation $M_{C}\left(V_{G}\right)$ ($M_{C}$ is related to the conductance of the contact region) is clearly dependent on the annealing temperature (see Figure 9). Note that, both in the as-prepared and 150 °C-annealed samples, $M_{C}\left(V_{G}\right)$ does not follow the trend given in Equation (12), but in the 50 °C- and 100 °C-annealed samples the trend in Equation (12) is fulfilled. This indicates that defects or traps in the as-prepared sample can be irregularly distributed between the contact region and the intrinsic channel; they can be redistributed uniformly, or some of them even eliminated, if ${\mathrm{NiPc}}_{6}$ OTFTs are annealed. However, if the annealing temperature surpasses the value of 132 °C, corresponding to one of the thermally induced melts seen in Figure 2, new irregularities appear near the contact region. Note that an ${\mathrm{NiPc}}_{6}$ OTFT annealed at 150 °C undergoes a first transition of crystal to columnar mesophase (132 °C) when heated, and a subsequent re-crystallization at 96 °C when the annealed transistors are cooled.

The evolution of the parameter $m_{k}$ supports this conclusion. The value $m_{k}≃2$ in the as-prepared sample indicates a space-charge conduction through the contact. The value $m_{k}≃1$ in the 50 °C-annealed sample indicates that the transport is Ohmic through the contact. By increasing the annealing temperature space-charge conduction through the contact appears again ($m_{k}≃2$ at 150 °C). Despite the transformation of the contact region observed after the analysis of its characteristic parameters, note that the contact voltage decreases monotonically when increasing the annealing temperature. This benefits the overall performance of the ${\mathrm{NiPc}}_{6}$ transistor.

This analysis of the evolution of the relation $M_{C}\left(V_{G}\right)$ and the parameter $m_{k}$ with the annealing temperature is consistent with the evolution of the threshold voltage with the annealing temperature (Figure 7b). The increment of the annealing temperature results in a large number of carriers that are transported along the active channel, increasing $V_{T}$ to more positive values. However, this trend is broken at 150 °C, again just above the first induced melt detected at 132 °C (Figure 2).

The evolution of the carrier mobility with the annealing temperature, shown in Figure 8, indicates that increasing the annealing temperature, without approaching the transition of crystal to columnar mesophase, also favors the increment of the mobility. This is the case of 50 °C. In the case of 150 °C, the samples have overcome the transition of crystal to columnar mesophase when annealed and the recrystallization when cooled. The changes produced in these 150 °C-annealed OTFTs make the mobility recover approximately the value of the as-prepared case. The value of the carrier mobility of the 100 °C-annealed OTFTs is located between the ones of the 50 and 150 °C cases in almost the whole range of $V_{GT}$, $(|V_{GT}|>15\;V$, clearly in on-state, in which all the output characteristics were measured). Below this range the mobility is almost the same for the as-prepared, 100 and 150 °C cases. These results show that annealing the samples increases the value of the mobility, and the transition of crystal to columnar mesophase reverses this trend. Thus, an optimum temperature for the mobility is necessarily below the transition of crystal to columnar mesophase. In this experiment the position of this optimum temperature is around 50 °C. More measurements of $I_{D}-V_{D}$ curves at different temperatures (below and above 50 °C) would be necessary if a more precise value of the optimum annealing temperature was required. 

The electrical changes in the carrier mobility (which include the changes of $\mu_{o}$, $V_{T}$ and γ) are also consistent with the ATM images. On the one hand, the electrical changes observed in the OTFTs that were annealed below the crystal-to-columnar mesophase transition are consistent with the larger crystals seen in the ATM images. The highest value of the mobility, and more particularly the lowest value of γ detected at 50 °C, point out a more ordered semiconductor. Actually, a low value of γ is a typical feature of a semiconductor with a crystalline-like behavior and a narrow distribution of the DOS, which are consistent with the larger size of the crystals. On the other hand, the AFM images that correspond to the OTFTs annealed in the mesophase show the fluidity of the mesophase, and likely molecular re-alignment, with large liquid crystal domain formation (the large “discs” are domains). Recrystallization then takes place within the individual domains. As such, the crystals from this preparation regime are significantly smaller than the two lower temperature anneals. There will be boundaries between the larger domains, but also within them between crystallites. Thus, the disorder in the semiconductor is enhanced, which is reflected with an increment of the value of γ and a reduction of the value of the mobility in almost the whole $V_{GT}$ range (|$V_{GT}$| > 15 V).

As mentioned in Section 2.2, a similar study was done with annealed ${\mathrm{PdPc}}_{6}$-based OTFTs [63]. In that work, the transistors were analyzed following a similar method as the one proposed in this work, except that the evolutionary parameter extraction procedure is used in the present case. In both ${\mathrm{PdPc}}_{6}$- and ${\mathrm{NiPc}}_{6}$-based transistors, the increment of the annealing temperature over the first thermally induced melt, corresponding to the crystal-to-columnar mesophase transition, make the annealed samples deteriorate. In the ${\mathrm{PdPc}}_{6}$ case, the contact voltage increased when the annealing temperature was above this transition temperature, and thus made the drain current diminish, despite that the carrier mobility and the threshold voltage were monotonically increasing with the annealing temperature. In the ${\mathrm{NiPc}}_{6}$ case, the drain current and the contact voltage improve over the transition temperature although our parameter extraction procedure detects that the carrier mobility and threshold voltage decrease over this transition temperature.

## 6. Conclusions

A precise analysis of the effect of annealing on ${\mathrm{NiPc}}_{6}$ transistors has been carried out thanks to the combined interplay of DSC and AFM techniques and an electrical characterization. This electrical characterization has been supported by a generic compact model for OTFTs, which allows for a functional description of the contact effects, and an evolutionary parameter extraction procedure. The combination of all these techniques has been essential to determine the optimum range of annealing temperature to guarantee the best performance of the OTFTs.

Actually, increasing the annealing temperature improves the performance of the ${\mathrm{NiPc}}_{6}$ transistors. However, care must be taken if the annealing temperature approaches the value in which the transition of crystal to columnar mesophase takes place (132 °C). Above this temperature, the threshold voltage and the carrier mobility deteriorate. Damage is also detected in the contact region of the transistor when annealing above this temperature. However, the voltage drop at the contact region remains decreasing and the drain current also increases.

## Figures and Tables

**Table 1:** Parameters of the ${\mathrm{NiPc}}_{6}$ OTFTs.

Parameter	Samples (Annealing Temperature, °C)				Extracted From
	As-Prepared	50	100	150	
Average particle size (μm)	100 ± 10	200 ± 20	400 ± 20	1500 ± 100	AFM data
Surface roughness (nm)	1.52	17.80	42.62	65.19	AFM data
μ_0_ (cm^2^/Vs)	2.30 × 10^−4^	2.16 × 10^−3^	4.93 × 10^−5^	1.29 × 10^−4^	(5), (18), (20)
*γ*	0.402	0.116	0.971	0.619	(5), (18), (20)
$V_{T}$ (V)	4.18 × 10^−7^	5.90	14.5	12.1	(5), (18), (20)
$\hat{V_{T}}$ (V)	-	6.91	13.1	-	(10)
α ^[A/V(mk+1+γ]^	-	1.08 × 10^−10^	1.81 × 10^−12^	-	(10)
V_SS_ (V)	−8.88	−3.41	−20.4	−21.2	(5), (18), (20)
m_k_	1.98	1.10	1.64	2.00	(5), (18), (20)
M_C_(−20 V) [A/V^mk^]	1.48 × 10^−9^	4.21 × 10^−9^	1.90 × 10^−9^	8.91 × 10^−9^	(5), (18), (20)
M_C_(−30 V) [A/V^mk^]	1.04 × 10^−9^	6.38 × 10^−9^	2.80 × 10^−9^	4.47 × 10^−8^	(5), (18), (20)
M_C_(−40 V) [A/V^mk^]	6.82 × 10^−10^	7.72 × 10^−9^	4.62 × 10^−9^	4.96 × 10^−8^	(5), (18), (20)
M_C_(−50 V) [A/V^mk^]	6.46 × 10^−10^	1.00 × 10^−8^	6.48 × 10^−9^	5.70 × 10^−8^	(5), (18), (20)
